# Evaluation of Whole-Exome Enrichment Solutions: Lessons from the High-End of the Short-Read Sequencing Scale

**DOI:** 10.3390/jcm9113656

**Published:** 2020-11-13

**Authors:** Ana Díaz-de Usera, Jose M. Lorenzo-Salazar, Luis A. Rubio-Rodríguez, Adrián Muñoz-Barrera, Beatriz Guillen-Guio, Itahisa Marcelino-Rodríguez, Víctor García-Olivares, Alejandro Mendoza-Alvarez, Almudena Corrales, Antonio Íñigo-Campos, Rafaela González-Montelongo, Carlos Flores

**Affiliations:** 1 Genomics Division, Instituto Tecnológico y de Energías Renovables (ITER), 38600 Santa Cruz de Tenerife, Spain; adiaz@iter.es (A.D.-d.U.); jlorenzo@iter.es (J.M.L.-S.); lrubio@iter.es (L.A.R.-R.); amunoz@iter.es (A.M.-B.); vgarcia@iter.es (V.G.-O.); ainigo@iter.es (A.Í.-C.); rgonzalezmontelongo@iter.es (R.G.-M.); 2 Research Unit, Hospital Universitario N.S. de Candelaria, Universidad de La Laguna, 38010 Santa Cruz de Tenerife, Spain; bguillenguio@gmail.com (B.G.-G.); itahisa@gmail.com (I.M.-R.); amendoal@ull.edu.es (A.M.-A.); acorrales@fciisc.es (A.C.); 3 Instituto de Tecnologías Biomédicas (ITB), Universidad de La Laguna, 38200 San Cristóbal de La Laguna, Spain; 4 CIBER de Enfermedades Respiratorias, Instituto de Salud Carlos III, 28029 Madrid, Spain

**Keywords:** next-generation sequencing, whole-exome sequencing, target coverage, duplicate reads, genetic variation

## Abstract

Whole-exome sequencing has become a popular technique in research and clinical settings, assisting in disease diagnosis and increasing the understanding of disease pathogenesis. In this study, we aimed to compare common enrichment capture solutions available in the market. Peripheral blood-purified DNA samples were enriched with SureSelect^QXT^ V6 (Agilent) and various Illumina solutions: TruSeq DNA Nano, TruSeq DNA Exome, Nextera DNA Exome, and Illumina DNA Prep with Enrichment, and sequenced on a HiSeq 4000. We found that their percentage of duplicate reads was as much as 2 times higher than previously reported values for the previous HiSeq series. SureSelect^QXT^ and Illumina DNA Prep with Enrichment showed the best average on-target coverage, which improved when off-target regions were included. At high coverage levels and in shared bases, these two solutions and TruSeq DNA Exome provided three of the best performances. With respect to the number of small variants detected, SureSelect^QXT^ presented the lowest number of detected variants in target regions. When off-target regions were considered, its ability equalized to other solutions. Our results show SureSelect^QXT^ and Illumina DNA Prep with Enrichment to be the best enrichment capture solutions.

## 1. Introduction

Each of the steps involved in DNA sequencing has evolved to reduce the hands-on time, increase automation and versatility, and improve upon previous solutions [1]. Genomics has developed dramatically since the first next-generation sequencing (NGS) system was released in 2005 [2]. The introduction of NGS almost entirely displaced other alternatives for the analysis of genetic variation and has become an essential approach to use genomics at unprecedented levels. It has opened new research horizons and profoundly accelerated and changed how genetic studies are conducted and diseases are diagnosed [3,4,5].

For genetic disease studies, three main NGS approaches can be currently considered: targeted sequencing (TS) of a panel of genes, whole-exome sequencing (WES), and whole-genome sequencing (WGS). Although TS has obvious benefits by focusing only on the genes or regions of interest, reducing the ethical problems linked to the identification of incidental (secondary) findings, it has the drawback that the target is limited by current disease knowledge, which does not accommodate data reanalysis with new disease gene discoveries [6]. This is one of the main reasons why WES has gained popularity in the last years [4], evidencing clear benefits for gene discovery across many diseases, including intellectual disability [7], inflammatory bowel disease [8], epilepsy [5,7], and a broad range of Mendelian conditions [5,9,10,11,12,13]. All these studies highlight the improvement in the diagnostic yield, reporting diagnostic yields between 25 and 30% in some cases, and, more relevantly, the proper implementation of treatment of genetic disease and improvements in patient health outcomes thanks to WES based on short-read sequencing (SRS). Long-read sequencing (LRS) has emerged as a promising sequencing technique that allows avoiding the use of PCR or potential errors derived from technical manipulations, among others [14,15]. The higher percentage of read error per base and the lower throughput [6], linked to the need for high base accuracy in the clinical context, have motivated the better positioning of SRS in clinical settings. Although WGS has decreased its costs in recent years [16], its higher costs for routine testing, concerns due to the increase in findings of uncertain significance, and associated computational difficulties (e.g., increased data storage necessities) have fostered the widespread use of WES as a common approach in biomedical research. The most important reason explaining this swift spread is that exome, which represents approximately 1–2% of the genome, includes ~85% of all described disease-causing variants [17]. Over the last decade, sequencing chemistry and sequencing systems have developed meteorically, launching multiple sequencing systems that have improved different features such as cost-effectiveness, high-throughput, and production scale. At the high-end of the throughput scale of Illumina, the platforms combine sequencing by synthesis (SBS) chemistry with exclusion amplification (also known as ExAmp chemistry) and a patterned flow cell technology [18,19]. Given the lack of published benchmarks for sequencing platforms combining such peculiarities for WES, we aimed to assess a wide array of in-solution and bead-based enrichment capture protocols.

## 2. Experimental Section

### 2.1. Samples

DNA samples were obtained from peripheral blood using a commercial column-based solution (GE Healthcare, Chicago, IL, USA) from unrelated donors of European descent after informed consent. The study was approved by the Research Ethics Committee of the hospital (PI-07/12) and performed according to The Code of Ethics of the World Medical Association (Declaration of Helsinki). Quality controls (QCs) were performed on the 4200 TapeStation system using the Genomic DNA ScreenTape Assay (Agilent Technologies, Santa Clara, CA, USA) and Qubit^®^ 3.0 Fluorometer by the Qubit^TM^ dsDNA HS Assay Kit (Thermo Fisher Scientific, Waltham, MA, USA).

### 2.2. Enrichment Protocols

We focused on the following whole-exome enrichment solutions following the manufacturer’s recommendations for library preparation:

1. SureSelect^QXT^ Target Enrichment for Illumina Multiplexed Sequencing, V6 (Agilent Technologies, Santa Clara, CA, USA), Protocol version D1, December 2016. Five independent samples were processed with this solution. Fifty nanograms of genomic DNA (gDNA) was fragmented to a 150 base pair (bp) insert size. Adaptors were added in a single enzymatic step and were, subsequently, amplified (8 cycles). Next, up to 750 ng of each library were hybridized with the capture probes, and the capture was performed using streptavidin-coated beads. A postcapture PCR amplification (10 cycles) was carried out to add two index tags per sample.
2. TruSeq Nano DNA Library Prep, currently known as TruSeq DNA Nano (Illumina Inc., San Diego, CA, USA), Reference Guide protocol of June 2015. Five independent samples were processed with this solution. A total of 100 ng of gDNA was sheared by sonication to 350 bp size fragments on an M220 Focused-ultrasonicator (Covaris, Woburn, MA, USA), followed by end-repair, adenylation of 3′ end, and ligation to the specific adapter, which included a unique index. Libraries were amplified with Illumina primers (8 cycles), and 500 ng of each library was pooled in a single tube before the capture. At this point, the TruSeq Nano DNA protocol was continued as that of the Nextera DNA Exome (Illumina Inc.) protocol, performing two consecutive hybridizations. Finally, a postcapture PCR amplification (10 cycles) was carried out.
3. TruSeq Exome Library Prep, currently known as TruSeq DNA Exome (Illumina Inc.), Reference Guide protocol of November 2015. Five independent samples were processed with this solution. gDNA (100 ng) was sheared using the M220 Focused-ultrasonicator (Covaris). Fragments of 150 bp were end-repaired, adenylated on 3′ end, and ligated to the specific adapter, which included one index per sample. A precapture PCR amplification (8 cycles) was carried out previously to pooling libraries (100 ng) followed by two consecutive hybridizations. Finally, a postcapture PCR amplification (8 cycles) was carried out.
4. TruSeq Rapid Exome, currently known as Nextera DNA Exome (Illumina Inc.), Reference Guide protocol of December 2016. gDNA (50 ng) was fragmented by enzymatic digestion, and the adaptors were ligated simultaneously. Libraries were then amplified (10 cycles) with Illumina primers to add two indexes for each sample. Once labeled, libraries were pooled. Next, two consecutive hybridizations were performed to capture hybridized probes to the targeted regions of interest, with streptavidin magnetic beads. At the end, a postcapture PCR amplification (10 cycles) was carried out. This capture was tested under three different conditions in five independent samples for each one: (1) the standard 125 bp insert size; (2) 350 bp insert size; and (3) 450 bp insert size.
5. Nextera Flex for Enrichment, currently known as Illumina DNA Prep with Enrichment (Illumina Inc.), Reference Guide protocol of October 2018. Five independent samples were processed with this solution. Enrichment-bead-linked transposons (eBLT) were used to tagment 50 ng of gDNA and attach adapter sequences to the fragments. After eBLT clean-up, the addition of two indexes per sample by PCR amplification (9 cycles) was performed. Subsequently, 500 ng of each library were pooled for a single hybridization reaction and capture. The last step consisted of a postcapture PCR amplification (10 cycles).

The main differences between the enrichment protocols are summarized in Table 1. Regarding target size, SureSelect^QXT^ provided a target region of 60.5 Megabases (Mb), whereas all other tested enrichments targeted 45.3 Mb of the human genome. Because of this, we considered SureSelect^QXT^ as the reference for all comparisons. It is common to extend the target exonic regions to the flanking sequences by adding padding as part of the analysis pipeline. We considered two situations in the comparisons (strict and padding). For the comparison with padding, we imposed an extension of 100 bp on each side of exons, increasing the total target size for that comparison to 100.7 Mb for SureSelect^QXT^ and 85.4 Mb for the Illumina solutions.

### 2.3. Sequencing

Libraries were assessed by Qubit^TM^ dsDNA HS Assay Kit on Qubit^®^ 3.0 Fluorometer (Thermo Fisher Scientific) and the Agilent D1000 and High Sensitivity D1000 ScreenTape Assays on the 4200 TapeStation system (Agilent Technologies). Pools of indexed samples at 2 nM loading concentration were sequenced on an Illumina HiSeq 4000 Sequencing System (Illumina Inc.) with 75 bp paired-end reads, along with 1% of PhiX Control V3 (Illumina Inc.) according to the manufacturer’s instructions. Sequencing experiments were conducted at the Instituto Tecnológico y de Energías Renovables (Santa Cruz de Tenerife, Spain).

### 2.4. Bioinformatic and Statistical Analysis

**Figure 1:**
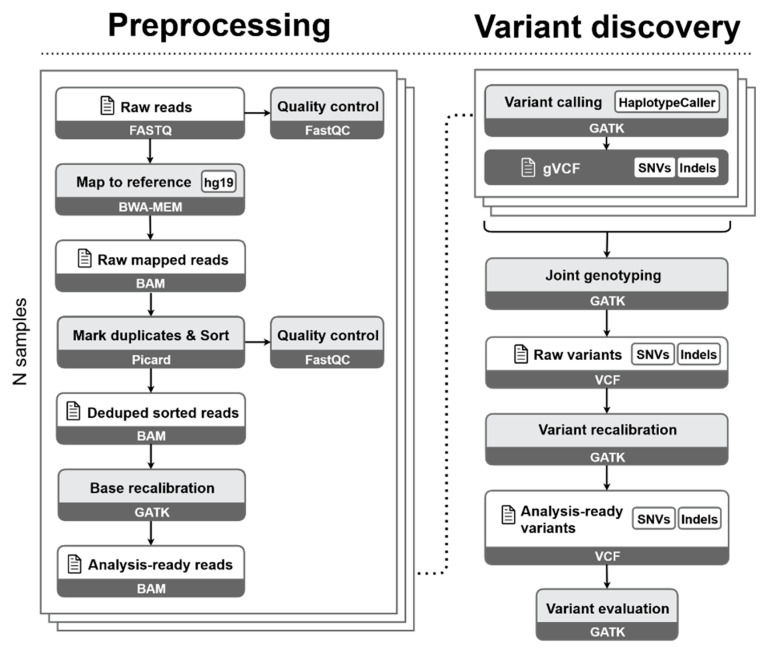
Schematic representation of the pipeline steps involved in the bioinformatic analysis. BWA-MEM: Burrows-Wheeler Aligner with Maximal Exact Matches algorithm. BAM: Binary Alignment Map. GATK: Genome Analysis Toolkit. gVCF: genomic Variant Calling Format. SNVs: single-nucleotide variants. Indels: insertions and deletions. VCF: Variant Calling Format.

The raw sequencing data were first normalized by downsampling with seqtk v. 1.3 [20] using the same random seed for all enrichment protocols. Genomic data were then processed on the TeideHPC Supercomputing facility (http://teidehpc.iter.es/en) using an in-house bioinformatics pipeline based on the Genome Analysis Toolkit (GATK) v. 3.8 Best Practices guidelines for short germline variant discovery (single-nucleotide variants (SNVs) and small insertions and deletions (indels)) [21]. The pipeline consisted of two stages (Figure 1): preprocessing and variant discovery.

An initial assessment of raw FASTQ reads was performed using FastQC v. 0.11.8 [22]. In the preprocessing stage, demultiplexed reads with trimmed adapter sequences from each sample were aligned to the reference genome (GRCh37/hg19) using the Burrows-Wheeler Aligner with Maximal Exact Matches algorithm (BWA-MEM) v. 0.7.15 [23]. Duplicate reads were marked, and alignments were sorted using Picard v. 2.1.1 [24]. Base quality score recalibration was performed by GATK. QC operations were carried out on the aligned reads prior to base score recalibration with Qualimap v.2.2.1 [25]. In the variant discovery stage, identification of single-nucleotide variants (SNVs) and indels was conducted using GATK HaplotypeCaller in the genomic Variant Calling Format (gVCF) mode. This result was recalibrated and refined using GATK Variant Quality Score Recalibration (VQSR) to distinguish variant calls that are likely to be true discoveries from those that are likely to be false. The final variant callset was evaluated using Picard CollectVariantCallingMetrics and GATK VariantEval by comparing relevant metrics between our results and the Single Nucleotide Polymorphism Database (dbSNP) build 138 known truth set, producing a final analysis-ready VCF (Figure 1). The resulting callset of each sample was combined into a single multisample VCF with refined variants for ulterior imputation. Manifest files, including target regions, were obtained from manufacturers of each exome capture solution.

Statistical differences among the enrichment protocol metrics were assessed in the R 4.0.2 environment considering pairwise comparisons against SureSelect^QXT^ V6, which was considered as the reference, based on the nonparametric Mann–Whitney U-test.

### 2.5. Genotype Imputation

Genotype imputation allows estimating missing genotypes based on variants supplied by VCF and aligned to reference panels by means of different software algorithms. This approach helps to find novel risk alleles in genome-wide association studies (GWAS) [26,27], as well as the increase in the likelihood of identifying causal variants thanks to higher-resolution data [28]. While this approach is widespread in GWAS, the possibilities of imputing variation from WES data is expected and will be necessary for standardizing the datasets when combined with traditional array-based GWAS studies. The Michigan Imputation Server [29], based on Minimac4, was used to assess the variant imputation capability of the different exome capture protocols. For this purpose, only SNVs, such as input data, were considered, given that indels usually result in poor call rates and genotype accuracy and lower imputation quality [30]. Imputation was based on reference data from Europeans from the Haplotype Reference Consortium (HRC) r1.1. 2016 panel using Eagle v. 2.4 for phasing. For simplicity, as a gross estimate, we count variants reaching an imputation quality threshold (Rsq) > 0.3, irrespective of the minor allele frequency.

### 2.6. Data Availability

The data that support the findings of this study are available on request from the corresponding author. The genotype and sequence data are not publicly available because of privacy or ethical restrictions.

## 3. Results

### 3.1. Alignment and Duplicates

Based on the raw data obtained, the passing filter (PF) bases and aligned PF bases showed high variability between enrichment protocols, ranging from a mean (±SD) of 4.45 ± 0.58 to 10.61 ± 0.55 Gbases. To normalize the comparisons, we first randomly downsampled all the reads to the lowest sequencing yield (i.e., 45.3 M reads) while keeping a 25% increment in the proportion of reads for the SureSelect V6 enrichment protocol to adjust for its larger target size.

**Figure 2:**
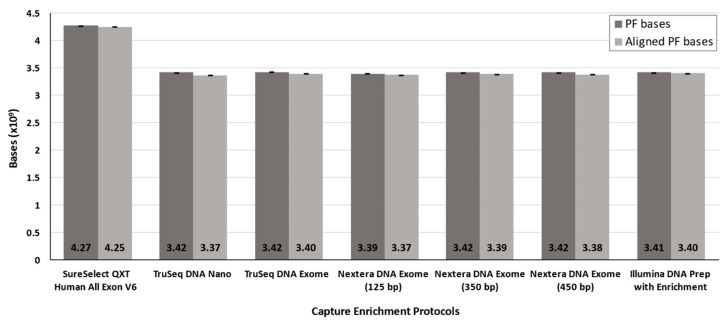
Average number of bases passing the filters and aligned bases after downsampling. Bars represent average ± standard deviation. PF: passing filter. bp: base pair.

Basic sequencing data after downsampling data is provided in Table A1. On average, between 98.58% ± 0.05 and 99.56% ± 0.02 of the bases passing the filters aligned against the reference genome in the enrichment solutions, regardless of using strict or padding as the targets (Figure 2).

**Figure 3:**
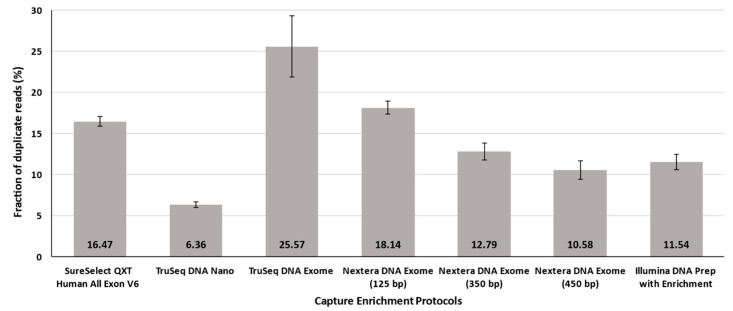
Duplicate reads across solutions. Bars represent average ± standard deviation.

The average number of observed duplicate reads for all enrichment protocols deserves a special mention at this point, since TruSeq DNA Exome showed much larger proportions in the HiSeq 4000 instrument than those declared by the manufacturer specifications (Figure 3). TruSeq DNA Exome and, strikingly, the Illumina standard for Nextera DNA Exome-125 bp provided the highest proportion among all tested protocols, with 25.57% ± 3.73 and 18.14% ± 0.80, respectively. The lowest percentages of duplicates were obtained for TruSeq DNA Nano (6.36% ± 0.33) and Nextera DNA Exome-450 bp (10.58% ± 1.13) (Figure 3).

### 3.2. Guanine-Cytosine Bias

**Figure 4:**
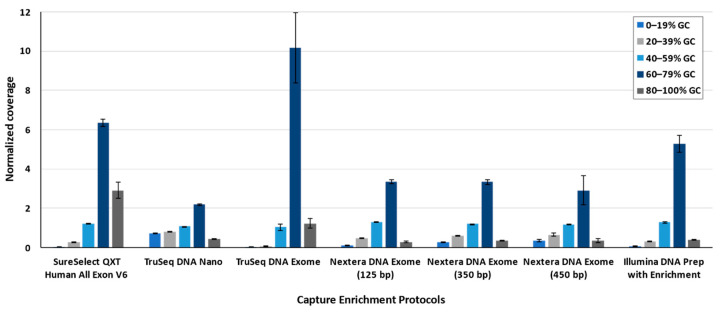
Normalized coverage based on GC content per range. Normalized coverage represents how different are the coverages per range of GC content with respect to the mean total coverage. Bars represent average ± standard deviation.

The guanine-cytosine (GC) bias was evaluated for the different enrichment protocols by default parameters of the Picard Tool, considering both strict and padding conditions. The five quintiles of the content in the GC percentage showed a common pattern among all solutions: the quintiles corresponding to the low percentage of GC content (<40%) have significantly lower coverage than the mean total coverage (Table A2). On the other hand, for the quintiles with high proportions of GC content (>60–79%), the coverage increased up to 10.18 (±1.78) times above the mean coverage. In this comparison, TruSeq DNA Exome had an outlier behavior for the quintiles with high proportions of GC content (Figure 4).

### 3.3. Target Coverage

**Figure 5:**
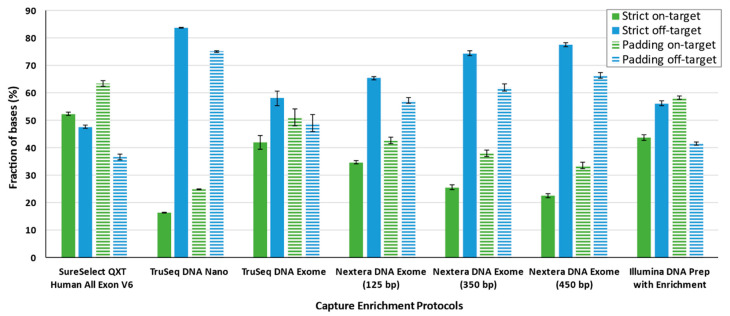
On- and off-target fraction of bases on average. Bars represent average ± standard deviation.

Coverage metrics were calculated both for on-target and off-target regions, as well as considering the targets strictly or with padding. The best average on-target coverage was obtained for SureSelect^QXT^ V6 (strict: 52.37% ± 0.55; padding: 63.40% ± 1.02), whereas TruSeq DNA Nano showed the lowest coverage (strict: 16.30% ± 0.14; padding: 24.93% ± 0.19) (Table 2). Among these two extremes, Illumina DNA Prep with Enrichment and TruSeq DNA Exome provided the second and third best on-target coverages, respectively. Among the Nextera DNA Exome protocols, the protocol with a 125 bp insert size was optimal for strict and padding conditions. For the strict target, SureSelect^QXT^ V6 from Agilent and Illumina DNA Prep with Enrichment and TruSeq DNA Exome from Illumina were the best enrichment capture solutions. Padded analyses provided the larger proportion of on-target coverage and, consequently, the lowest off-target. Under the padding condition, SureSelect^QXT^ V6, Illumina DNA Prep with Enrichment, and TruSeq DNA Exome were the only solutions in which the proportion of bases in on-target regions was higher than those off-target (Figure 5).

**Figure 6:**
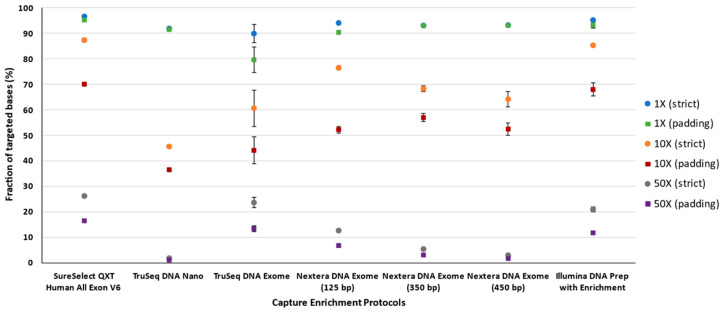
Average fraction of targeted bases at 1, 10, and 50×. Bars represent average ± standard deviation.

The fraction of the target that was covered at a given depth varied widely among the protocols, particularly at a large depth of coverage. There were no substantial differences if the target was considered strictly or with padding at 1× depth of coverage. The TruSeq DNA Exome protocol constituted a clear exception, as it showed a substantial decrease in the percentage of targeted bases (Figure 6). Considering the strict target and at 10× depth of coverage, the order from best to worst was SureSelect^QXT^ V6, Illumina DNA Prep with Enrichment, Nextera DNA Exome-125 bp, Nextera DNA Exome-350 bp, Nextera DNA Exome-450 bp, TruSeq DNA Exome, and TruSeq DNA Nano. When padding was considered in the analysis, a similar scenario was observed, except that Nextera DNA Exome-125 bp ranked lower. At 50× depth of coverage, the protocols with the largest insert sizes showed the worst depth of coverage under any situation, closely followed by TruSeq DNA Nano.

**Figure 7:**
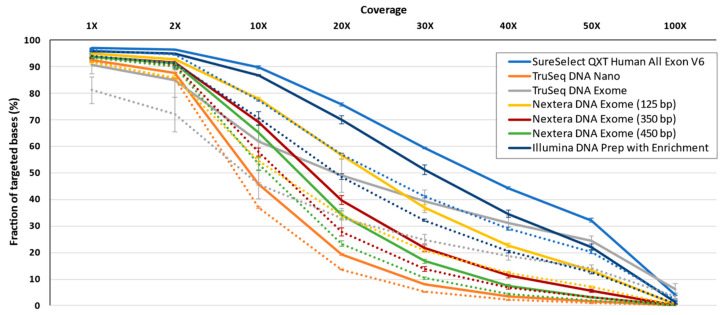
Average fraction of coverage at 1, 2, 10, 20, 30, 40, 50 and 100× on shared regions across enrichment solutions. Strict target condition in solid line; padding condition in dotted line. Lines represent average ± standard deviation.

The tested technologies have differences, including the target territory. We were interested in assessing the different vendors and protocols when the same (shared) regions are considered. For this end, all bases shared between the technologies were analyzed, resulting in 39.6 Mb for the strict and 80.7 Mb for the padding targets. Regardless of padding, a clear pattern was observed. For a depth of coverage below 2X, all protocols brought an expected excellent performance, with the exception of TruSeq DNA Exome and TruSeq DNA Nano, which had the worst performances. At depths larger than 10×, SureSelect^QXT^ V6, and Illumina DNA Prep with Enrichment provided the best results, being more obvious when the analysis focused strictly on target (Figure 7). Nextera DNA Exome-125 bp presented a good performance up to 20× depth of coverage in the strict target analysis and up to 10× when padding was used (Figure 7). Strikingly, TruSeq DNA Exome provided one of the most gradual decreases in the fraction of targeted bases as the covered fraction values increased. However, for coverages above 40×, it showed one of the best results. When the padding was considered, its performance decreased perceptibly.

### 3.4. Ability to Detect SNVs and Small Indels

An average of 70,020 ± 9239 SNVs and small indel variants was called using the strict target region, and 96,237 ± 17,852 when padding was included in the analysis, considering all samples for all protocols as a whole (Table 3). Regarding the strict target, we observed the largest number of variants with the Illumina DNA Prep with Enrichment protocol, although not far from those observed for other Illumina protocols. The results were similar when padding was incorporated into the analyses. However, in that case, the Nextera DNA Exome-450 bp was the protocol providing the largest number of variants while TruSeq DNA Exome had an outlier behavior on the lower end.

**Figure 8:**
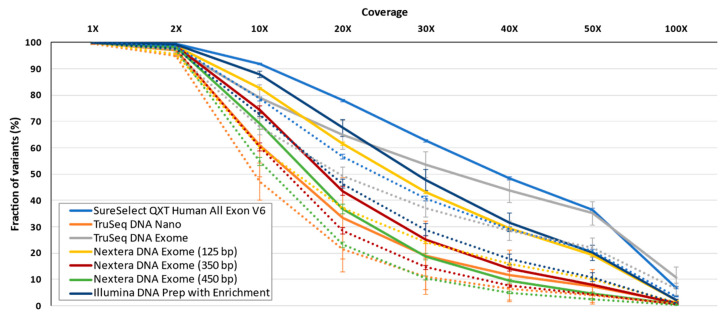
Average fraction of variants with varying depth of coverage on the shared regions across enrichment solutions. Strict target condition indicated by a solid line; padding condition indicated by a dotted line. Lines represent average ± standard deviation.

When comparing called variants from the 39.6 Mb of the shared target region (80.7 Mb using padding) for all samples and protocols, an average of 29,719 ± 1678 variants (69,832 ± 8162 variants with padding) was observed. Irrespective of padding, the same outlier behavior of the TruSeq DNA Exome was observed, showing the lowest number of called variants. Given that we did not use any internal control sample to assess the reproducibility of calls across enrichment solutions, we ten compared each solution with itself by calculating the ratio between the number of variants detected using padding and the number of variants using the strict condition. The rationale for this ratio is based on the notion that a significant proportion of reads obtained by exome sequencing map outside targets and, therefore, are usually ignored despite them allowing the detection of variants that provide information of interest for the disease [31], particularly those located in the vicinity of exons. Ultimately, this ratio informs about the gain of captured variants by off-target reads in the vicinity of exons for each enrichment protocol. The ratio padding/strict was highest for SureSelect^QXT^ V6, Nextera DNA Exome-350 bp. and Nextera DNA Exome-450 bp, whereas the lowest ratio was obtained for TruSeq DNA Exome (Table 4). Regardless of padding, SureSelect^QXT^ V6, closely followed by TruSeq DNA Exome and Illumina DNA Prep with Enrichment, offered the largest fraction of SNVs and indel variants covered under a wide range of coverages when only shared bases among protocols were considered (Figure 8).

### 3.5. Imputation Performance

When the strict target region was considered, the Illumina DNA Prep with Enrichment protocol showed the best performance for the imputation of uncovered variants (2.7 M), whereas the TruSeq DNA Nano protocol behaved the worst solution for imputation (2.4 M) (Table 5). Under the padding condition, the Nextera DNA Exome-350 bp protocol had the best performance (3.0 M), while the TruSeq DNA Exome protocol provided the lowest number of imputed variants (2.4 M).

## 4. Discussion

This study constitutes a broad assessment of marketed whole-exome capture solutions when paired with short-read sequencing obtained with ExAmp chemistry and patterned flow cells. For that, we focused on Illumina and Agilent technologies, given their widespread use in clinical settings and accessibility and evaluating seven different protocols. Our observations support that SureSelect^QXT^ V6 and Illumina DNA Prep with Enrichment kits are among the optimal solutions offering the most robust results as well as the best relationship between performance and turnaround time. A summary of all assessments and a qualitative comparison of all solutions are summarized in Table 6 for simplicity and guidance. We warn that these assessments lack an evaluation of true positives, false positives, or false negatives in the variant calls as our study did not include a control sample that was systematically analyzed across the different enrichment protocols.

SureSelect^QXT^ V6 was the only method assessed based on adjacent RNA probes for the capture, albeit baits with overlapping regions are more desirable than on adjacent or gapped baits [32]. Agilent improves hybridization and enrichment efficiency by relying on RNA baits with more extended probe sizes for better tolerance of hybridization mismatches [33] due to the greater binding strength between RNA–DNA heteroduplexes. On the other hand, the recent commercialization of the Illumina DNA Prep with Enrichment kit uses magnetic bead-based library preparation in the replacement of solution-based library preparation, allowing DNA tagmentation and adapter-ligation to occur in a single step. This, and the possibility to accommodate a wide range of input material, make this kit very attractive, increasing its versatility and efficiency [34]. The use of a constant amount of eBLT, regardless of the experiment, provides improved control in the normalization of the obtained material and consistency in tight fragment size distributions. This is because only the genomic DNA that attaches to the bead-based transposome complex is tagmented and adapter-ligated. Therefore, only this fraction is subjected to all ulterior steps in the protocol. These features could underlie its improved performance over all other Illumina protocols that were assessed, making it comparable to the SureSelect^QXT^ V6 solution.

In the study, the TruSeq DNA Nano and TruSeq DNA Exome solutions provided the worst results overall. Curiously, they both have two appealing features that are not provided by any other tested solution. The starting genomic DNA is 100 ng, which could be considered as a good starting point due to a higher initial amount, since it is usually related to improved library complexity [35]. On the other hand, mechanical fragmentation is the method of choice for DNA fragmentation in TruSeq DNA Nano and TruSeq DNA Exome solutions, whose performances were poorer when compared to the other exome enrichment alternatives assessed. However, the need for such quantities of input material may also be a limitation in some settings (e.g., with formalin-fixed paraffin-embedded tissue samples) because not all samples could yield such an input material [36]. The need for additional complementary equipment and the ultrasonication-based fragmentation step might also be considered as a drawback if high-throughput library preparation is to be pursued. Another issue was related to the outlier behavior of TruSeq DNA Exome regarding the GC bias. The GC bias varies among the different library protocols and sequencing platforms [37], constituting a typical issue derived from NGS. The main reasons to explain the uneven GC coverage could be either the inherent bias of the PCR amplification [38] or reduced efficiency of the capture probe hybridization [39]. However, since all Illumina protocols evaluated present the same probeset, it is unlikely that the GC bias is caused by the inefficient capture of the target region. Despite that, it is important to remark that TruSeq DNA Exome involves a longer hybridization time. 

In short-read sequencing data obtained with ExAmp chemistry and patterned flow cells, duplicate reads are a major issue and several types of them can be observed. On one side, biological duplication occurs randomly when two identical fragments were produced by DNA fragmentation, whereas the more problematic duplicates are generated in the PCR step [40] during library preparation. Optical and ExAmp duplicates are also generated during the sequencing process [41,42]. Optical duplicates are defined if the distance between the flow cell coordinates leading to two reads is within a 2500-pixel distance set by Picard’s *OPTICAL_DUPLICATE_PIXEL_DISTANCE* parameter. In an attempt to reduce these, newer Illumina platforms use patterned flow cells to collect the cluster data into nanowells that are sufficiently separated. With their introduction, the ExAmp duplicates emerged. For now, this issue has only been reported for HiSeq 3000/4000, HiSeq X Five and Ten Systems, and the NovaSeq series, as a consequence of seeding neighboring nanowells with identical fragments while amplification is running [43]. As a result, the rise of duplicate reads is not trivial in these platforms. As we have shown in this study, for TruSeq DNA Exome solutions, the increase is almost 2 times the number of average duplicates compared to kit specifications for assessed vendors (4–15%) [32,44]. As a matter of fact, three of the five standard protocols tested in the current study provided the highest proportion of duplicate reads, evidencing that most current whole-exome capture protocols are ill-adapted to the most novel Illumina platforms. Regarding ExAmp duplicates, they occur because the same library can seed one nanowell and is free to go back into the solution and reseed other close nanowells. A way to reduce this second seed might be through balancing between the number of polyclonal clusters and the percentage of duplicates. The higher the loading concentration, the lower the duplicate reads percentage at the cost of a higher proportion of polyclonal clusters [45]. Although the number of reads passing filters is much higher for the newest platforms than that obtained for the previous models, it is up to the users if this performance compensates the penalty for the very high percentage of duplicates observed.

Despite that SureSelect^QXT^ V6 was the tested solution with the highest target territory and provided the best results for the target bases (1–50×) in the strict mode, it showed the lowest number of detected variants. However, when padding was used in the analysis, SureSelect^QXT^ V6 duplicated its variant detection capability to a level comparable to the rest of the whole-exome capture solutions tested, irrespective of focusing only on shared regions across them. This fact highlights the nontrivial number of variants that the whole-exome captures allow to keep out of target territories [32,46]. Off-target reads, which could represent 40–60% of the reads in a WES study [47,48,49], can also be a source of informative genetic variation, as was evidenced by the gains (ratios) between those detected using padding vs. the strict condition. This was less evident for the TruSeq DNA Exome solution and was interpreted as an indication that many of its off-target sequencing reads were not informative of the variation in flanking exons at a near distance but likely more sparsely distributed in the genome. In agreement with this, although based on an expanded target region, this solution was previously suggested to have a major weakness by the high proportion of off-target reads [50] and that many of these reads map >200 bp away from the enrichment targets [48]. Although exome regions include ~85% of all described disease-causing variants [17], the rest of the genome contains functional elements such as UTRs in 3′ and 5′, silencers, or enhancers, which are vital in the regulatory process [51,52,53,54] and in the expression of complex disorders [55,56]. In this way, the National Human Genome Research Institute (NHGRI) has been developing the Encyclopedia of DNA Elements (also known as ENCODE project) [57] since 2007 to provide a catalog of functional elements in the human and mouse genomes. Multiple studies have pinpointed the utility of including off-target reads in NGS for routine analysis because they allow discovering genotype variants across the genome at low coverage (1–2X) [58,59] and genotyping common variants at a minimal depth (0.2–0.5×), albeit with high error rates [60]. Wang and colleagues [61] pointed out the importance of including low-priority regions (i.e., off-target reads or bases in WES analysis) in the context of disease association studies [62]. According to their study, these by-products could also be used to estimate genetic ancestry even with extremely low coverage (0.001× for worldwide continental ancestry and 0.10× for European ancestry). Therefore, off-target reads, which are commonly removed from the analysis of WES, could be an attractive source of information that may be worth considering. In this respect, genotype imputation analysis and subsequent association studies are usually carried out in array-based approaches. However, in the last decade, a flourishing of studies relied on NGS technologies considering off-target regions in the analyses [63,64]. Our results support this strategy as there was a clear increase in the number of imputed variants, for example in SureSelect^QXT^ V6, when off-target bases were considered in the inference.

## 5. Conclusions

With the rapid adoption of sequencing technologies in the last decade in clinical settings and in multidisciplinary research, diverse whole-exome capture solutions have emerged in the market. This study was intended to serve as evidence-based guidance based on the performance comparison among some of the most extended whole-exome capture solutions. Despite that the use of reference samples would have been desirable to provide complementary information (i.e., the precision of the variant callset), we opted to analyze samples drawn from unrelated donors from the same population. We conclude that, among the tested alternatives, SureSelect^QXT^ V6 and Illumina DNA Prep with Enrichment demonstrated the most robust results.

## Figures and Tables

**Table 1:** Main features of the exome enrichment solutions evaluated.

Features	SureS V6	TS DNA Na	TS DNA Ex	Nxt DNA Ex (125 bp)	Nxt DNA Ex (350 bp)	Nxt DNA Ex (450 bp)	Ill DNA Enr
Library prep	SB	SB	SB	SB	SB	SB	BB
Oligo probes	RNA	DNA	DNA	DNA	DNA	DNA	DNA
Tiling	Adjacent	Gapped	Gapped	Gapped	Gapped	Gapped	Gapped
Target size (Mb)	60.5	45.3	45.3	45.3	45.3	45.3	45.3
Input (ng)	50	100	100	50	50	50	50 ^a^
Fragmentation	Tagm	Ultrasont	Ultrasont	Tagm	Tagm	Tagm	Tagm
Insert size (bp)	150	350	150	125	350	450	150
Enrichment	Prepool	Postpool	Postpool	Postpool	Postpool	Postpool	Postpool
Time (days)	1	3	4	3	3	3	2
Hybridization time (min)	79	40 + 40 ^b^	118 + 898 ^b^	40 + 40 ^b^	40 + 40 ^b^	40 + 40 ^b^	114
Cost per sample	***	**	*	**	**	**	**

^a^ Specifications indicate that this can range from 10 ng to 1 μg, depending on the type of the starting material (i.e., blood, saliva, FFPE, etc.). ^b^ Two capture steps. Relative costs are indicated with categories, where the price correlates positively with the number of indicated asterisks (* means inexpensive, ** means intermediate cost, and *** means the most expensive). SureS V6: SureSelect^QXT^ Human All Exon V6, TS DNA Na: TruSeq DNA Nano, TS DNA Ex: TruSeq DNA Exome, Nxt DNA Ex: Nextera DNA Exome, Ill DNA Enr: Illumina DNA Prep with Enrichment. bp: base pair. SB: solution-based, BB: bead-based, Tagm: tagmentation, Ultrasont: ultrasonication.

**Table 2:** On-target coverage for the exome enrichment protocols.

On-Target Bases (%)	SureS V6	TS DNA Na	TS DNA Ex	Nxt DNA Ex (125 bp)	Nxt DNA Ex (350 bp)	Nxt DNA Ex (450 bp)	Ill DNA Enr
Strict target	52.37 ± 0.55	16.30 ± 0.14 **	41.98 ± 2.58 **	34.65 ± 0.61 **	25.59 ± 0.79 **	22.46 ± 0.80 **	43.79 ± 0.99 **
Padding	63.40 ± 1.02	24.93 ± 0.19 **	51.07 ± 3.15 **	42.66 ± 1.05 **	38.00 ± 1.23 **	33.61 ± 1.11 **	58.36 ± 0.60 **

SureS V6: SureSelect^QXT^ Human All Exon V6, TS DNA Na: TruSeq DNA Nano, TS DNA Ex: TruSeq DNA Exome, Nxt DNA Ex: Nextera DNA Exome, Ill DNA Enr: Illumina DNA Prep with Enrichment. bp: base pair. Numbers refer to average ± standard deviation. Statistical significance of the differences between enrichment protocols compared to SureSelect^QXT^ V6 indicated as: ^ns^ *p* > 0.05; * *p* ≤ 0.05; and ** *p* ≤ 0.01.

**Table 3:** Summary of called variants.

Total Variants	SureS V6	TS DNA Na	TS DNA Ex	Nxt DNA Ex (125 bp)	Nxt DNA Ex (350 bp)	Nxt DNA Ex (450 bp)	Ill DNA Enr
Strict target	54,418 ± 681	68,401 ± 1118 **	65,247 ± 4410 **	66,592 ± 1603 **	75,309 ± 917 **	76,809 ± 3206 **	83,362 ± 4984 **
Padding	111,588 ±1326	98,100 ± 602 **	68,750 ± 5408 **	72,422 ± 2016 **	110,479 ± 1172 ^ns^	112,823 ± 2568 ^ns^	99,494 ± 10,120 **

SureS V6: SureSelect^QXT^ Human All Exon V6, TS DNA Na: TruSeq DNA Nano, TS DNA Ex: TruSeq DNA Exome, Nxt DNA Ex: Nextera DNA Exome, Ill DNA Enr: Illumina DNA Prep with Enrichment. bp: base pair. Numbers refer to average ± standard deviation. Statistical significance of the differences between enrichment protocols compared to SureSelect^QXT^ V6 indicated as: ^ns^ *p* > 0.05; * *p* ≤ 0.05; and ** *p* ≤ 0.01.

**Table 4:** Summary of detected variants on shared target regions across enrichment solutions.

Total Variants	SureS V6	TS DNA Na	TS DNA Ex	Nxt DNA Ex (125 bp)	Nxt DNA Ex (350 bp)	Nxt DNA Ex (450 bp)	Ill DNA Enr
Strict (39.6 Mb)	31,415 ± 329	28,258 ± 1279 **	27,680 ± 1700 **	30,297 ± 267 **	29,844 ± 266 **	30,569 ± 957 ^ns^	30,863 ± 413 ^ns^
Padding (80.7 Mb)	78,308 ± 618	67,359 ± 438 **	57,376 ± 4419 **	66,707 ± 1406 **	75,289 ± 920 **	76,578 ± 2378 ^ns^	74,750 ± 3507 *
Ratio padding/strict	2.49	2.38	2.07	2.20	2.52	2.51	2.42

SureS V6: SureSelect^QXT^ Human All Exon V6, TS DNA Na: TruSeq DNA Nano, TS DNA Ex: TruSeq DNA Exome, Nxt DNA Ex: Nextera DNA Exome, Ill DNA Enr: Illumina DNA Prep with Enrichment. bp: base pair. Numbers refer to average ± standard deviation. Statistical significance of the differences between enrichment protocols compared to SureSelect^QXT^ V6 indicated as: ^ns^ *p* > 0.05; * *p* ≤ 0.05; and ** *p* ≤ 0.01.

**Table 5:** Summary of imputed variants.

Imputed Variants	SureS V6	TS DNA Na	TS DNA Ex	Nxt DNA Ex (125 bp)	Nxt DNA Ex (350 bp)	Nxt DNA Ex (450 bp)	Ill DNA Enr
Strict target	2,486,956	2,391,563	2,392,600	2,421,952	2,669,902	2,621,444	2,748,015
Padding	2,887,442	2,690,742	2,398,783	2,450,524	2,960,721	2,891,555	2,771,099

SureS V6: SureSelect^QXT^ Human All Exon V6, TS DNA Na: TruSeq DNA Nano, TS DNA Ex: TruSeq DNA Exome, Nxt DNA Ex: Nextera DNA Exome, Ill DNA Enr: Illumina DNA Prep with Enrichment. bp: base pair. Numbers represent averages.

**Table 6:** Qualitative assessment of whole-exome enrichment solutions. Performance is schematically represented by a color scale, where green indicates excellent, pale green indicates very good, yellow indicates fair, and red indicates poor performance. bp: base pair.

		SureSelect^QXT^ V6	TruSeq DNA Nano	TruSeq DNA Exome	Nextera DNA Exome (125 bp)	Nextera DNA Exome (350 bp)	Nextera DNA Exome (450 bp)	Illumina DNA Prep with Enrichment
Library prep								
Oligo probes								
Tiling								
Target size (Mb)								
Input (ng)								
Fragmentation								
Enrichment								
Time (days)								
Hybridization time (min)								
Cost per sample								
Aligned PF bases								
Duplicates								
% on-target bases								
% targeted bases 1×								
% targeted bases 10×								
% targeted bases 50×								
% targeted shared bases	1×							
	10×							
	30×							
	40×							
	50×							
	100×							
Total variants (strict target)								
Total variants (padding)								
Total variants (in shared bases)								
Imputed variants (strict target)								
Imputed variants (padding)								

## Appendix A

**Table A1:** Summary of sequencing data after downsampling data.

Sequencing Data.	SureS V6	TS DNA Na	TS DNA Ex	Nxt DNA Ex (125 bp)	Nxt DNA Ex (350 bp)	Nxt DNA Ex (450 bp)	Ill DNA Enr
Total GBases ^a^	4.27 ± 0.0003	3.42 ± 0.0001	3.42 ± 0.001	3.39 ± 0.004	3.42 ± 0.0002	3.42 ± 0.001	3.41 ± 0.001
GBases in Q30 ^a^	4.01 ± 0.01	3.13 ± 0.01	3.32 ± 0.003	3.21 ± 0.01	3.13 ± 0.01	3.10 ± 0.02	3.23 ± 0.01
PF Mreads ^a^	56.59	45.27	45.27	45.27	45.27	45.27	45.27
PF Mreads aligned ^a^	56.50 ± 0.01	45.02 ± 0.02	45.10 ± 0.07	45.17 ± 0.02	45.14 ± 0.01	45.11 ± 0.02	45.23 ± 0.005
Coverage ^a^ Strict target	36.82 ± 27.49	12.10 ± 13.15	31.37 ± 36.83	25.82 ± 20.71	19.13 ± 16.34	16.74 ± 14.06	32.85 ± 23.49
Coverage ^a^ Padding	26.76 ± 25.84	9.83 ± 11.14	20.26 ± 30.61	16.87 ± 18.58	15.08 ± 14.14	13.30 ± 12.22	23.24 ± 21.58
Mapping Q ^b^ Strict target	56.89 ± 0.03	56.77 ± 0.04	56.51 ± 0.08	56.61 ± 0.03	56.71 ± 0.04	56.76 ± 0.01	56.68 ± 0.04
Mapping Q ^b^ Padding	56.83 ± 0.04	57.12 ± 0.04	56.73 ± 0.11	56.97 ± 0.04	57.08 ± 0.04	57.12 ± 0.01	57.03 ± 0.04
GC percentage ^b^ Strict target	54.13 ± 0.17	49.48 ± 0.09	58.67 ± 1.67	49.63 ± 0.24	50.27 ± 0.33	49.83 ± 1.44	52.49 ± 0.73
GC percentage ^b^ Padding	53.80 ± 0.25	47.33 ± 0.12	58.57 ± 1.76	48.93 ± 0.27	48.89 ± 0.39	48.34 ± 1.60	51.60 ± 0.65

SureS V6: SureSelect^QXT^ Human All Exon V6, TS DNA Na: TruSeq DNA Nano, TS DNA Ex: TruSeq DNA Exome, Nxt DNA Ex: Nextera DNA Exome, Ill DNA Enr: Illumina DNA Prep with Enrichment. bp: base pair. GBases: Gigabases. Q: Phred-like quality scores. PF: passing filter. Mreads: Megareads. GC: guanine-cytosine. Mapping Q: Mapping Quality. Numbers refer to average ± standard deviation. ^a^ Metrics from Picard Tools, ^b^ Metrics from Qualimap.

**Table A2:** GC bias distribution by quintiles (Q) among the exome enrichment protocols.

Quintiles	SureS V6	TS DNA Na	TS DNA Ex	Nxt DNA Ex (125 bp)	Nxt DNA Ex (350 bp)	Nxt DNA Ex (450 bp)	Ill DNA Enr
Q1 (0–19%)	0.04 ± 0.01	0.73 ± 0.02 **	0.02 ± 0.004 ^ns^	0.12 ± 0.01 **	0.29 ± 0.02 **	0.35 ± 0.06 **	0.08 ± 0.02 *
Q2 (20–39%)	0.26 ± 0.02	0.81 ± 0.01 **	0.06 ± 0.02 **	0.47 ± 0.01 **	0.58 ± 0.02 **	0.66 ± 0.08 **	0.31 ± 0.02 **
Q3 (40–59%)	1.22 ± 0.01	1.08 ± 0.01 **	1.04 ± 0.17 ^ns^	1.32 ± 0.01 **	1.20 ± 0.01 *	1.17 ± 0.01 **	1.29 ± 0.03 **
Q4 (60–89%)	6.36 ± 0.20	2.19 ± 0.04 **	10.18 ± 1.78 **	3.34 ± 0.11 **	3.35 ± 0.12 **	2.92 ± 0.73 **	5.28 ± 0.44 **
Q5 (90–100%)	2.91 ± 0.41	0.43 ± 0.03 **	1.22 ± 0.25 **	0.29 ± 0.03 **	0.36 ± 0.03 **	0.34 ± 0.10 **	0.40 ± 0.03 **

SureS V6: SureSelect^QXT^ Human All Exon V6, TS DNA Na: TruSeq DNA Nano, TS DNA Ex: TruSeq DNA Exome, Nxt DNA Ex: Nextera DNA Exome, Ill DNA Enr: Illumina DNA Prep with Enrichment. bp: base pair. Q: quintile. Numbers refer to the average ± standard deviation. Statistical significance of the differences between enrichment protocols compared to SureSelect^QXT^ V6 indicated as: ^ns^, *p* > 0.05; *, *p* ≤ 0.05; and **, *p* ≤ 0.01.
